# Supplementary material for: The Unfolded Protein Response Sensor PERK Mediates Stiffness-Dependent Adaptation in Glioblastoma Cells
Source: Int J Mol Sci. 2022 Jun 10;23(12):6520. doi: 10.3390/ijms23126520 (PMC9223606; doi:10.3390/ijms23126520)
Supplement: Supplementary file 1 [file ijms-23-06520-s001.zip › ijms-1754159-supplementary.pdf]

**Supplementary information**

## Rheology

*G*' was measured to be 1, 24 and 38 kPa at a frequency of 100 Hz for hydrogels with alginate concentration of 0.0, 0.84 and 1.81% w/v, respectively.

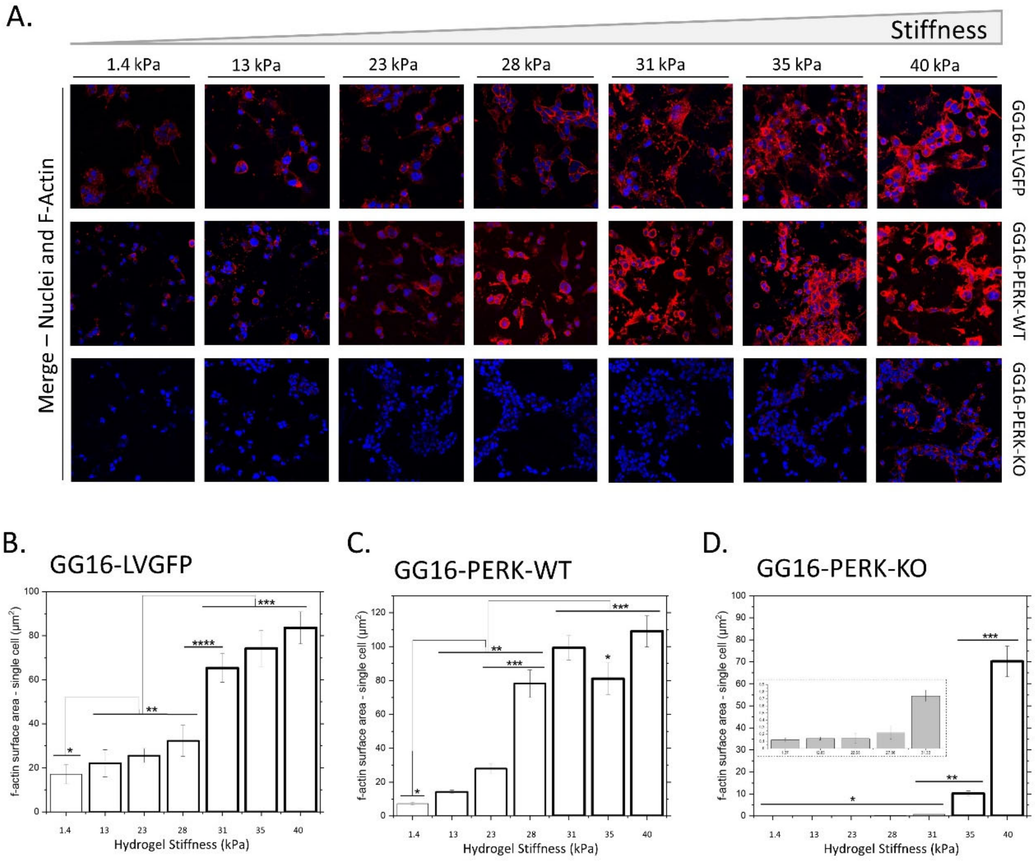


**Figure S1.** Cellular adaption of GG16 to matrix stiffness. GG16-LVGFP, GG16-WT and GG16-PERK-KO cells were cultured in wide range of stiffnesses (1.4 – 40 kPa) for 6 days. Cells were stained for F-Actin (red) and imaged by confocal microscopy (A). The F-Actin surface area was quantified at each matrix stiffness for all cell lines. Stiffness dependent increases in F-Actin surface areas is comparable for GG16-LVGFP and GG16-WT cells (B and C). However, PERK-KO cells show no or very low F-Actin levels and surface area and is only detected at the stiffest matrix (40 kPa) (A and D).

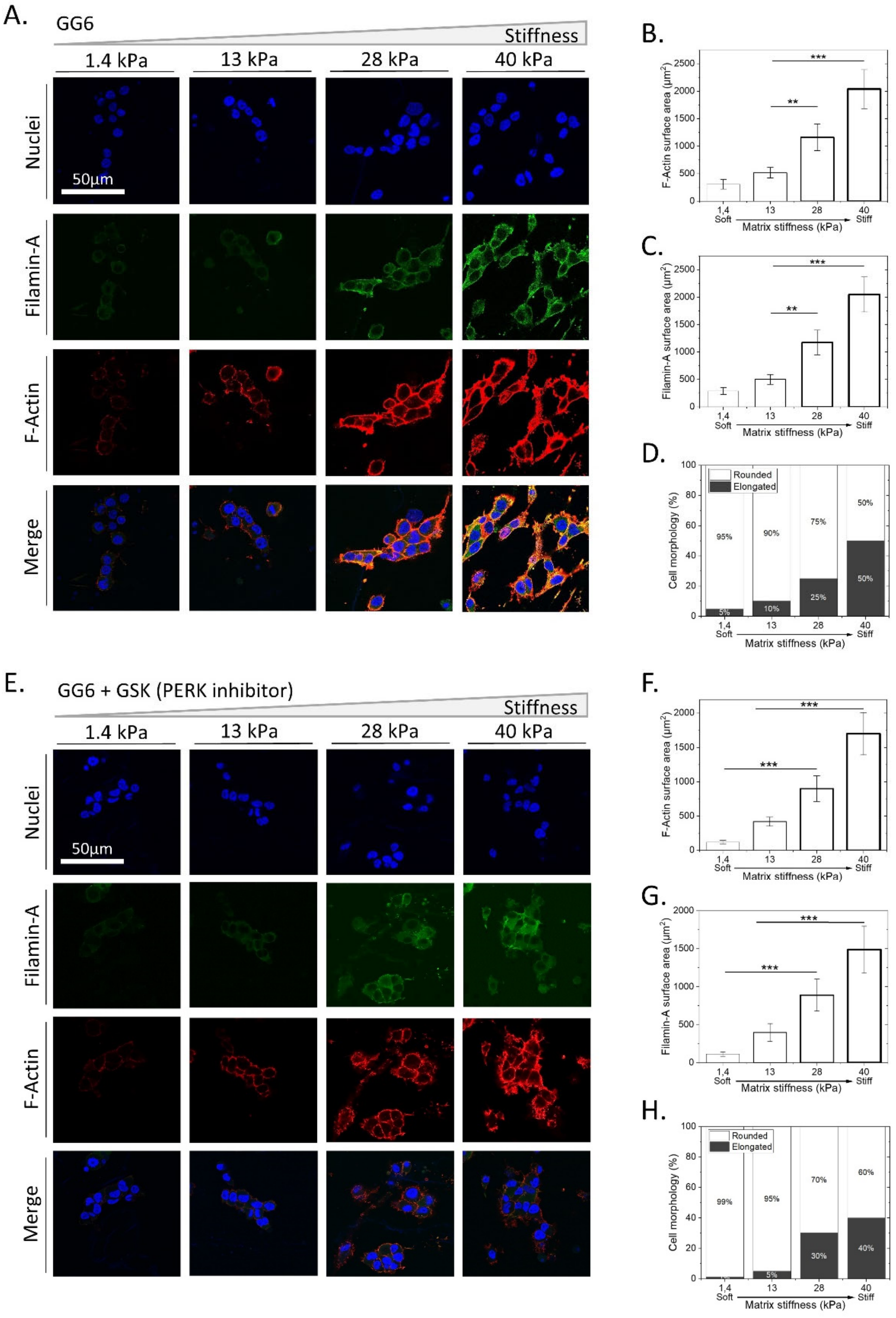


**Figure S2.** Cellular adaptation of GG6 cells to matrix stiffness. GG6 cells in absence or presence of GSK414 were cultured for 6 days on HBP/alginate hydrogels with different stiffnesses. (A and E) Cells were then stained for FLNA and F-Actin and imaged with confocal microscopy. FLNA and F-Actin surface area was measured for both GG6 and GG6 treated with GSK414 (B, C, F and G). GG6 showed similar as GG16 stiffness dependent increases in FLNA and F-Actin expression that was accompanied with cell elongation (D, H). GSK414 treatment

partially suppressed FLNA and F-Actin expression and cell elongation indicating that PERK kinase activity contributes to stiffness adaptation.

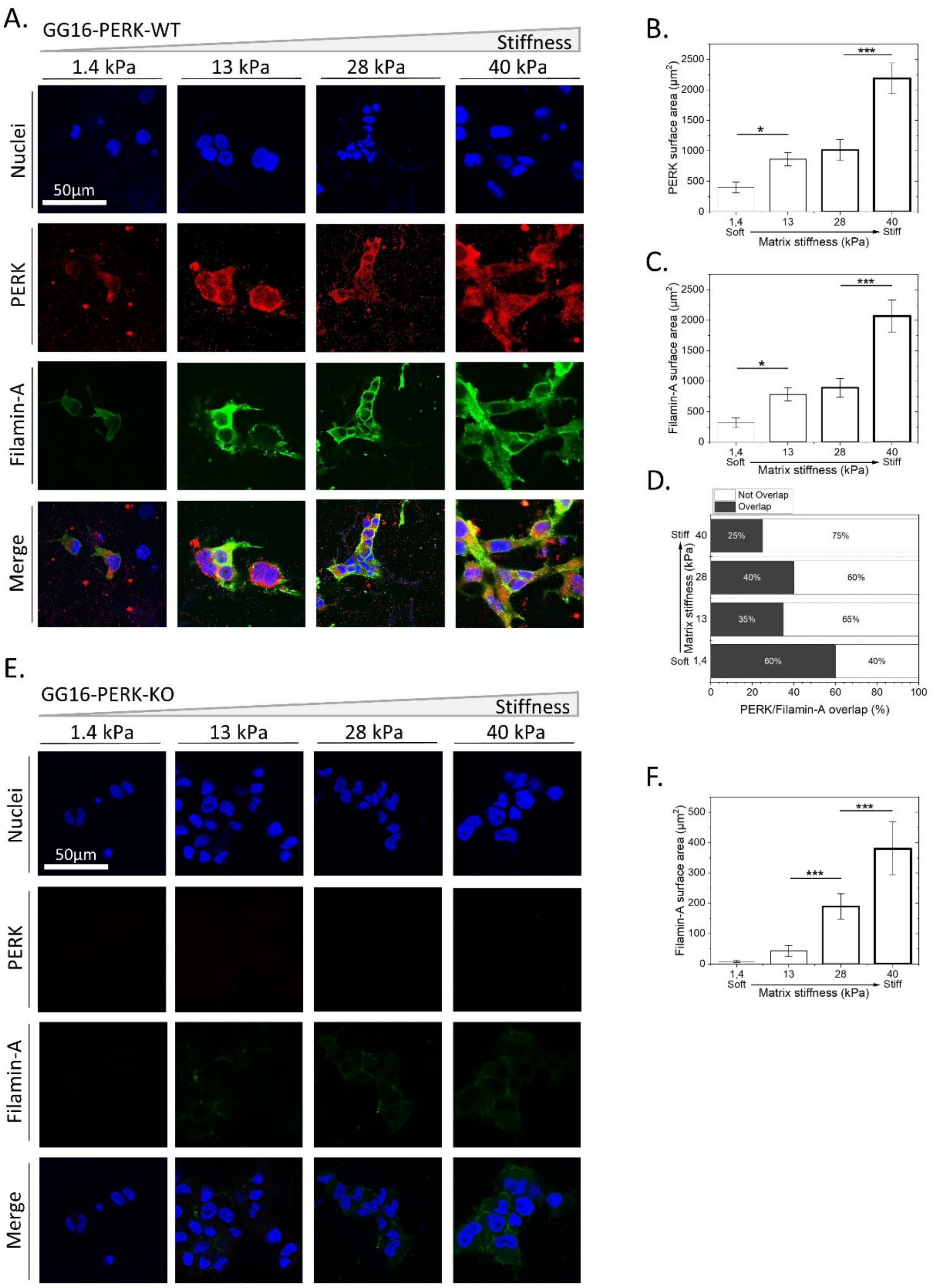


**Figure S3.** Stiffness-dependent increases in FLNA and PERK expression. GG16-WT and PERK-KO cells were cultured on hydrogels with increasing stiffnesses for 6 days. FLNA and PERK was determined by immunofluorescent microscopy (A and E). FLNA and PERK surface areas were measured (B and C). Stiffness dependent increases of both FLNA and PERK expression were

detected in GG16; FLNA and PERK expression partially overlapped (D). In PERK-KO cells, obviously no PERK was detected and FLNA expression was strongly reduced (E-F).
